# Distribution of Organic Carbon in the Sediments of Xinxue River and the Xinxue River Constructed Wetland, China

**DOI:** 10.1371/journal.pone.0134713

**Published:** 2015-07-31

**Authors:** Qingqing Cao, Renqing Wang, Haijie Zhang, Xiuli Ge, Jian Liu

**Affiliations:** 1 Institute of Environmental Research, Shandong University, Jinan 250100, China; 2 School of Life Sciences, Shandong University, Jinan 250100, China; 3 School of Environmental Science and Engineering, Qilu University of Technology, Jinan, 250353, China; DOE Pacific Northwest National Laboratory, UNITED STATES

## Abstract

Wetland ecosystems are represented as a significant reservoir of organic carbon and play an important role in mitigating the greenhouse effect. In order to compare the compositions and distribution of organic carbon in constructed and natural river wetlands, sediments from the Xinxue River Constructed Wetland and the Xinxue River, China, were sampled at two depths (0–15 cm and 15–25 cm) in both upstream and downstream locations. Three types of organic carbon were determined: light fraction organic carbon, heavy fraction organic carbon, and dissolved organic carbon. The results show that variations in light fraction organic carbon are significantly larger between upstream and downstream locations than they are between the two wetland types; however, the opposite trend is observed for the dissolved organic carbon. There are no significant differences in the distribution of heavy fraction organic carbon between the discrete variables (e.g., between the two depths, the two locations, or the two wetland types). However, there are significant cross-variable differences; for example, the distribution patterns of heavy fraction organic carbon between wetland types and depths, and between wetland types and locations. Correlation analysis reveals that light fraction organic carbon is positively associated with light fraction nitrogen in both wetlands, while heavy fraction organic carbon is associated with both heavy fraction nitrogen and the moisture content in the constructed wetland. The results of this study demonstrate that the constructed wetland, which has a relatively low background value of heavy fraction organic carbon, is gradually accumulating organic carbon of different types, with the level of accumulation dependent on the balance between carbon accumulation and carbon decomposition. In contrast, the river wetland has relatively stable levels of organic carbon.

## Introduction

Wetland ecosystems play an important environmental protection function, which includes the purification of waters, environmental governance, and the maintenance of socio-economic value [1–3]. As a significant reservoir of organic carbon (OC) [4, 5], wetland also help to mitigate the effects of climate change by offsetting the release of CO_2_ into the atmosphere [6, 7]. Globally, wetlands hold up to 40% of the OC budget (~1400 Pg C) [8], despite accounting for just 4–6% of the global land area [9, 10], and wetland carbon accumulation rate can reach 0.033 ± 0.0029 kg m^−2^ y^−1^ [11]. Although the storage of OC is accompanied by its mineralization [12], which is a process that can release CO_2_ and methane [13], carbon sequestration by wetlands remains a topic of significant interest [4, 5, 8].

Following carbon sequestration, most OC is stored as organic matter (OM) in soils and sediments [14]. The conditions under which carbon sequestration occurs can differ; for example, the morphological structure and density of OM in soils or wetland sediments varies during plant decay and microbial degradation [15–17]. The resulting separation of OM by density provides evidence regarding the distribution characteristics of OC in the matter cycle [18]. The light fraction OM (LFOM; density < = 1.7 g cm^−3^) is mainly representative of undecomposed or partially decomposed plant debris and microorganisms [19–21]. It is the main component of labile OC and is easily affected by turnover rate and climate change [22]. In most wetlands, LFOM accounts for a small proportion of the OC [21, 23] and can partially transform into heavy fraction OM (HFOM; density > = 1.7 g cm^−3^) during microbial degradation [16, 17]. HFOM, which is the primary constituent of OM in soils and wetlands [24, 25], forms as a collection of soil aggregates and particles, whose structure is complex but relatively stable [15, 26]. Besides, dissolved OM (DOM), which is mostly lost during the separation process, contains the dissolved OC (DOC) of sediments. DOC, which is also a major component of labile OC [27, 28], has a significant effect on bioavailability, and the physical and chemical processes in the wetland ecosystem [29]. In summary, the storage and composition of OC can be understood by determining light fraction OC (LFOC) from LFOM, heavy fraction OC (HFOC) from HFOM, and DOC from DOM.

Natural wetlands have naturally evolving relationships with the local environments [30], whereas constructed wetlands are built and managed in order to simulate natural wetlands [31–33] for purposes that include water purification and pollution abatement [34]. Carbon sequestration rates and the distribution of various types of OC can differ between the two wetlands. Understanding the behavior of OC in both constructed wetlands and river wetlands is important for the evaluation of the ecosystem and of the carbon cycle. For example, as most constructed wetlands have slower water velocities and higher vegetation coverage than natural river wetlands [35, 36], the characteristics of the constructed wetland may help to improve carbon sequestration. So, we hypothesize that the composition and distribution patterns of OC differ between constructed wetlands and the corresponding river wetlands. In order to test the proposed hypothesis, we analyze the spatial and vertical distributions of LFOC, HFOC, and DOC in the Xinxue River (XR) and the Xinxue River Constructed Wetland (XRCW), Shandong Province, China.

## Materials and Methods

### Ethics statement

The sample collection of our study was conducted with the official permission of the Environmental Protection Bureau of Weishan Country and the Xinxue River Constructed Wetland Management Committee.

### Site selection and field sampling

**Fig 1:**
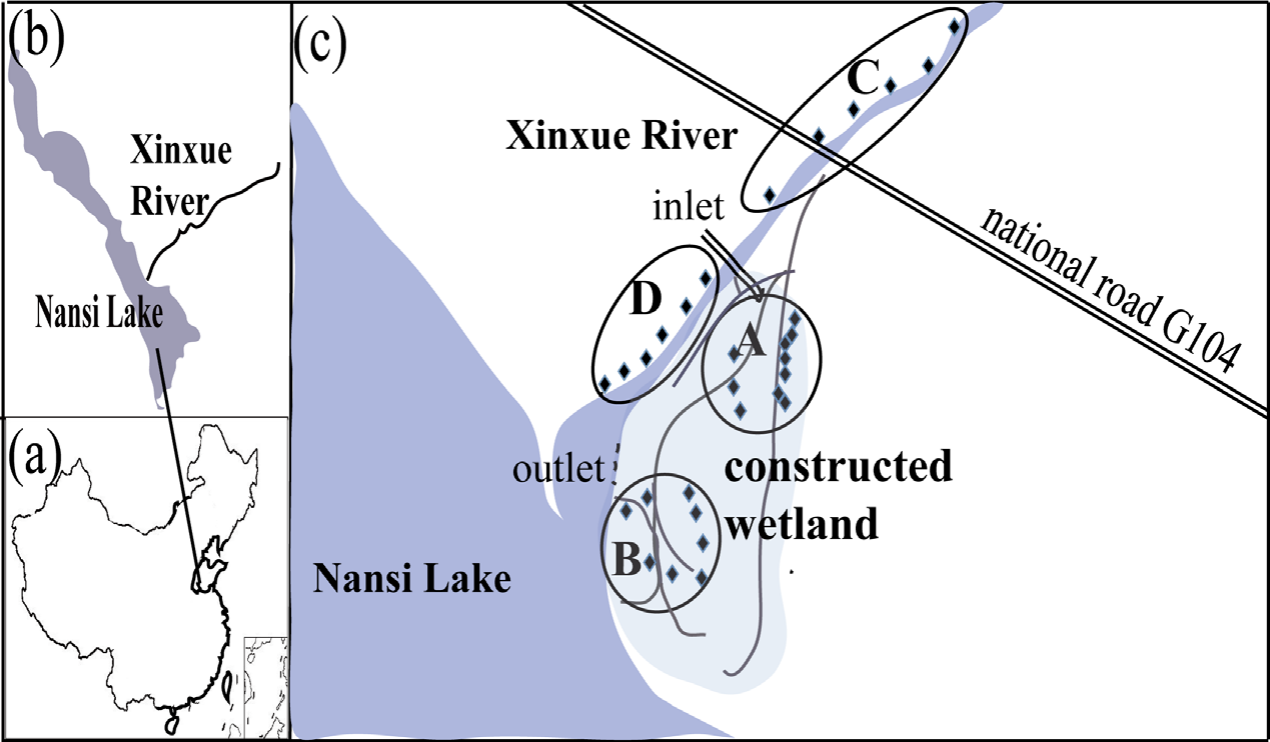
National, regional, and local maps of the study area. Maps showing the (a) national, (b) regional, and (c) local geographical setting of the study area. In map (c), showing the location of the Xinxue River (XR) and Xinxue River Constructed Wetland (XRCW): A = upstream location of XRCW; B = downstream location of XRCW; C = upstream location of XR; D = downstream location of XR.

This study was conducted at the XRCW and XR, Shandong Province, China (Fig 1a). XR is one of the principal rivers feeding Nansi Lake (Fig 1b), one of the biggest lakes in the South-to-North Water Diversion Project. The XRCW was constructed in 2007 in order to purify waters from XR before it flows into Nansi Lake and the XR estuary. Located on the southern bank of the XR, it is 5100 m long, 270 m wide, and covers an area of ~1000 m^2^ [37].

We collected a total of 60 sediment samples (30 samples from 0–15 cm in depth and 30 samples from 15–25 cm in depth) across four areas: upstream and downstream locations of the XRCW and natural XR wetland, respectively (Fig 1c). For the XRCW, 20 samples were collected from the upstream location (34°45’11.34” –34°45’51.45”N and 117°08’36.86” –117°08’58.09”E), while 16 samples were collected from the downstream location (34°43’58.96” –34°44’22.18”N and 117°09’06.83” –117°09’18.27”E). For the natural XR wetland, 12 samples were collected from both the upstream (34°46’26.80” –34°48’16.56”N and 117°08’59.40” –117°10’11.21”E) and downstream locations (34°45’14.10” –34°46’06.44”N and 117°08’29.52” –117°08’46.80”E).

Field sampling was conducted in October 2014. Sediment samples were collected using a sediment sampler and stored in sealed bags for transport to the laboratory. The moisture content and bulk density of all 60 samples were measured before the separation of OM.

### Laboratory analyses

Prior to further analysis, sediment samples were ground and then air dried at room temperature (~20°C). For the determination of DOC, samples were passed through a 2 mm sieve, while for LFOM and HFOM analyses, samples were prepared by passing through a 0.9 mm sieve.

The determination of DOC followed the methodology of Jones and Willett [38] and Dittmar et al. [39]. Sediment samples (10.00 g) were first mixed with KCl (2 M, 50 ml) in polyethylene bottles, and then shaken for 1 h at 20°C. Following filtration and centrifugation, DOC was measured by a total-C analyzer (TOC-L CPN, Shimadzu, Japan) using a non-purgeable OC analysis procedure. Blank tests were conducted to reduce experimental error. The pH of the filtrate was measured using a pH meter.

The contents of LFOC and HFOC were measured using the modified gravity method, as described by Zhang et al. [21]. Sediment samples were separated into light fractions (LF) and heavy fractions (HF) using 1.70 g mL^−1^ of sodium iodide solution. Centrifuge tubes containing 10.00 g samples and 40 ml sodium iodide solution were subjected to ultrasonic shaking for 10 min and then LF was filtered into 0.043 mm brass sieves. This operation was repeated 3 times to ensure complete separation. The LF was washed using 0.01 M calcium chloride solution and then distilled water, which was added to the LF until all Cl^-^ reactions ceased. The HF was washed 6 times by adding 0.01 M calcium chloride solution, stirring, centrifugation, and then decanting the suspensions until all I^-^ reactions ceased. Finally, distilled water was added to wash away the deposited material. The LF and HF were transferred into weighed beakers and then oven-dried at 60°C before being weighed. The contents of C and N were examined by an elemental analyzer (Vario EL III, Elementar Analysensysteme, Germany) and then LFOC, HFOC, light fraction nitrogen (LF-N), heavy fraction nitrogen (HF-N), and the carbon to nitrogen ratio of the light fraction (LF-C/N = LFOC/LF-N) and heavy fraction (HF-C/N = HFOC/HF-N) were calculated.

### Data analyses

Data analyses were performed using the SPSS (version 21.0) software. Two-way ANOVA were used to determine the interactions between the two depths and wetland types (the river and constructed wetlands), between two depths and locations (upstream and downstream of each wetland), and between wetland types and locations. When significant differences were observed, we used a Duncan test for post hoc multiple comparisons. One-way ANOVA were used to determine the significant differences between the two depths, two wetland types, and two locations because of the existence of interactions among them. Pearson correlation analysis was implemented among all the studied environmental factors.

## Results

### Distribution patterns of LFOC

**Fig 2:**
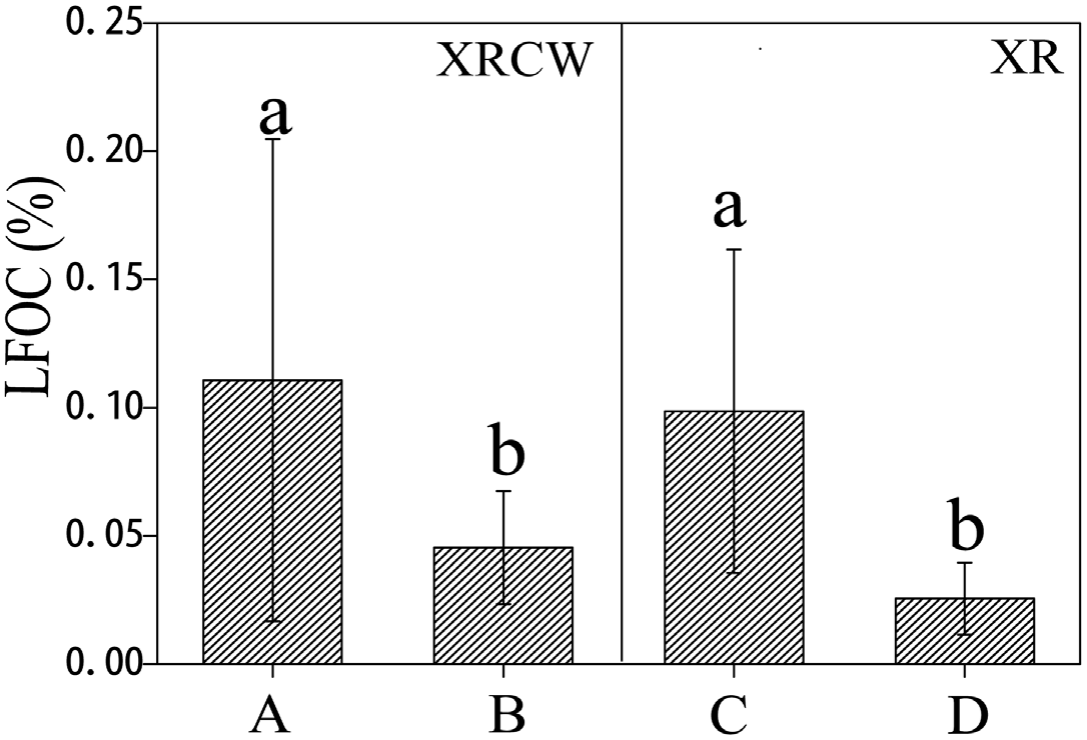
The spatial distribution of light fraction organic carbon (LFOC). Bar plots showing the spatial distribution of light fraction organic carbon (LFOC) in the Xinxue River (XR) and Xinxue River Constructed Wetland (XRCW). A = upstream location of XRCW; B = downstream location of XRCW; C = upstream location of XR; D = downstream location of XR. Bars sharing the same lowercase letter (a or b) are not significant at α = 0.05 (Duncan test).

The LFOC does not vary significantly between the two depth intervals (p = 0.432 for 0–15 cm and 15–25 cm; Table 1). However, LFOC levels vary significantly with locations (p = 0.000). LFOC is consistently higher in the upstream locations (0.099% in XR and 0.111% in XRCW) than in the corresponding downstream locations (0.025% in XR and 0.045% in XRCW; Fig 2; Table 2).

**Table 1:** Two-way analysis of different factors.

	Depths^a^	Locations^b^	Types^c^	Depths × Types	Depths × Locations	Types × Locations
LFOC	0.432	**0.000**	0.335	0.252	0.812	0.362
HFOC	0.554	0.707	0.574	**0.044**	**0.037**	0.820
DOC	0.933	**0.000**	**0.000**	0.760	**0.000**	0.914
N content	0.255	0.127	**0.000**	0.023	0.931	0.087
C/N	0.360	**0.004**	**0.000**	0.998	**0.020**	0.094
Moisture content	0.058	**0.006**	**0.000**	0.020	0.825	0.364
Bulk density	**0.011**	0.200	**0.001**	0.245	0.487	0.085
pH	0.796	0.116	**0.000**	0.859	**0.003**	0.576

^a^. Depths = 0–15 cm and 15–25 cm.

^b^. Locations = upstream and downstream locations.

^c^. Types = river wetland and constructed wetland.

Results with significant differences (*p* < 0.05) were shown in bold.

**Table 2:** The spatial distribution of OC types.

	Xinxue River Constructed Wetland			Xinxue River		
Mean (SD)	Upstream location	Downstream location	ALL	Upstream location	Downstream location	ALL
LFOC (%)	0.111 (0.094)	0.045 (0.022)	0.082 (0.078)	0.099 (0.063)	0.025 (0.014)	0.062 (0.058)
HFOC (%)	1.807 (0.595)	2.072 (0.710)	1.925 (0.653)	2.044 (0.562)	1.665 (0.366)	1.855 (0.503)
DOC (mg/L)	5.095 (2.910)	14.593 (3.066)	9.316 (5.616)	2.622 (0.859)	3.246 (1.093)	2.934 (1.013)

### Distribution patterns of HFOC

**Fig 3:**
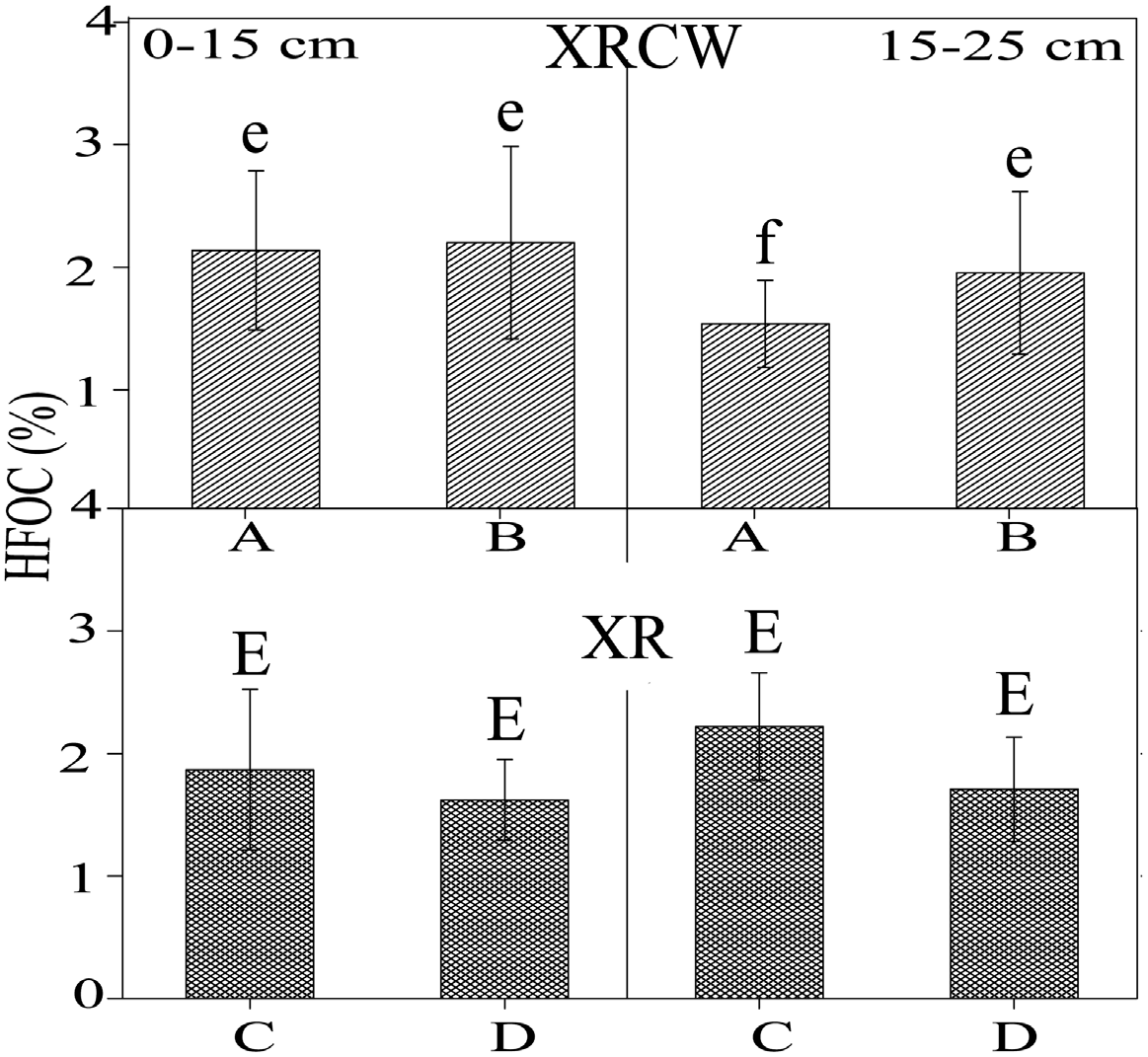
The spatial and vertical distribution of heavy fraction organic carbon (HFOC). Bar plots showing the spatial and vertical (0–15 cm vs. 15–25 cm) distribution of heavy fraction organic carbon (HFOC) in the Xinxue River (XR) and Xinxue River Constructed Wetland (XRCW). A = upstream location of XRCW; B = downstream location of XRCW; C = upstream location of XR; D = downstream location of XR. Bars sharing the same letter are not significant at α = 0.05 (Duncan Test).

There are no significant differences in the distribution of HFOC between the discrete variables (e.g., p = 0.554, 0.707, and 0.574 between the HFOC of the two depths, the two location types, and the two wetland types, respectively; Table 1); although mean HFOC in the XRCW (1.93%) is slightly higher than that in the XR wetland (1.86%; Table 2). However, significant cross-variable differences between wetland types and between the two depths show that HFOC in the upstream location of the XRCW is slightly–significant lower than in the downstream location, while for the XR wetland the opposite is true (Table 1; Fig 3). For example, at 15–25 cm, HFOC (1.97%) in the downstream location of the XRCW is significantly higher than in upstream location (1.51%). In contrast, at 0–15 cm, HFOC in the downstream location (2.17%) of the XRCW is higher than in upstream location (2.11%). The opposite trend is observed for the XR. For both wetland types, the differences are not significant at a depth of 0–15 cm. Differences are also observed between wetland types and locations, with HFOC values higher in the downstream location of XRCW (2.07%) than in the upstream location (1.81%). In contrast, for the XR, HFOC values are lower in the downstream location (1.67%) than in the upstream location (2.04%).

### Distribution patterns of DOC

**Fig 4:**
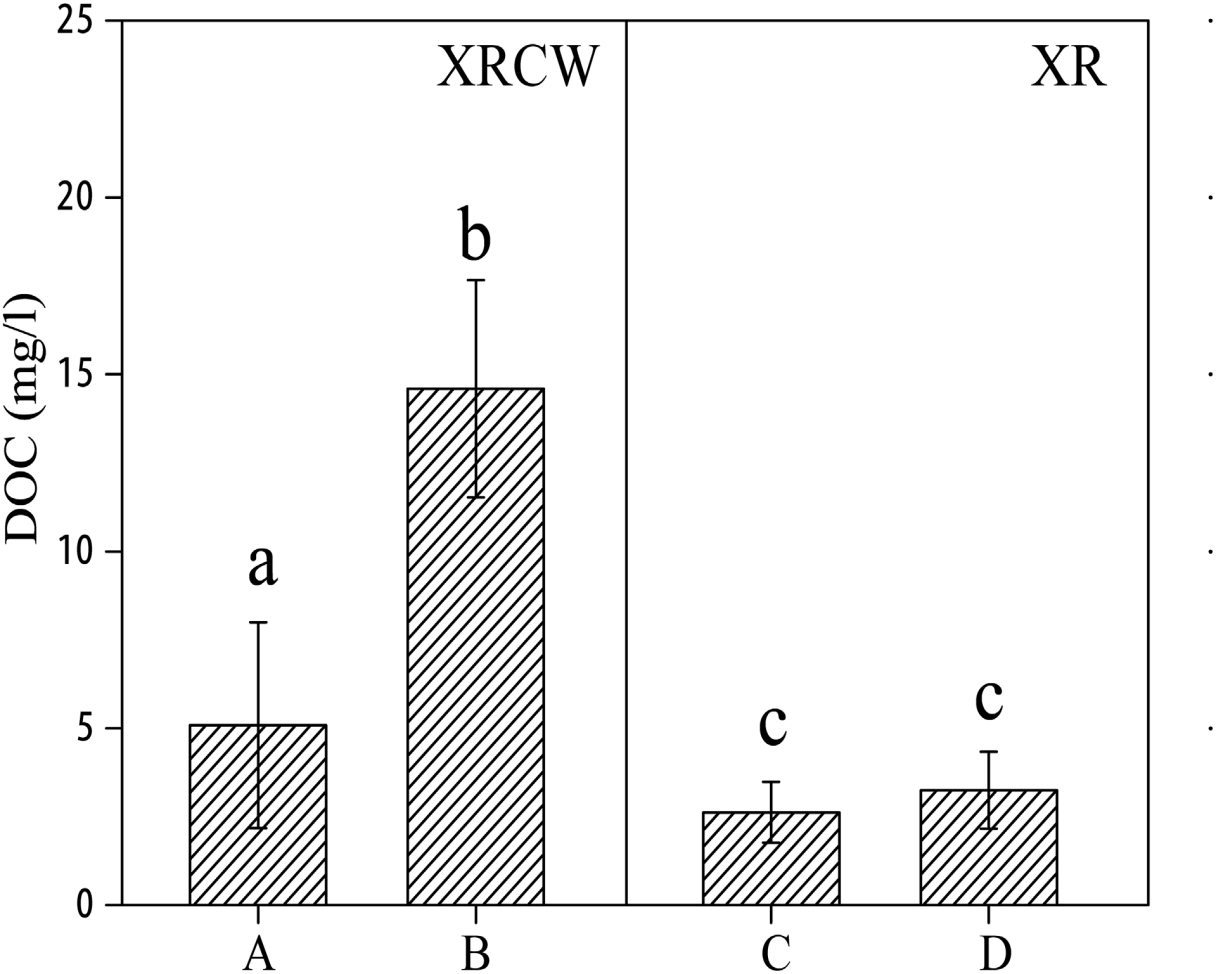
The spatial distribution of dissolved organic carbon (DOC). Bar plots showing the spatial distribution of dissolved organic carbon (DOC) in the Xinxue River (XR) and Xinxue River Constructed Wetland (XRCW). A = upstream location of XRCW; B = downstream location of XRCW; C = upstream location of XR; D = downstream location of XR. Bars sharing the same lowercase letter (a or b) are not significant at α = 0.05 (Duncan Test).

DOC, which varies from 1.27 mg/L to 21.20 mg/L, fluctuates more intensely than LFOC and HFOC. The distribution of DOC differs significantly with wetland types (2.93 mg/L in XR and 9.32 mg/L in XRCW; Table 2; Fig 4). The DOC in the upstream location of the XRCW (5.10 mg/L) is also significantly lower than that in downstream location of the XRCW (14.60 mg/L); however, inter-location differences are not significant for the XR (Fig 4). Despite the large fluctuations in DOC between the wetland types, DOC is not significantly different between the two depths.

### Correlations between C, N, and environment variables

The correlations between C, N, and other environment variables (e.g., moisture content, bulk density, pH) are different for the XR (Table 3) and the XRCW (Table 4). For the XRCW (Table 4), the LFOC, HFOC, DOC, LF-N, and HF-N are all significantly related to each other while HF-N is not significantly related to LFOC and HFOC (p = −0.363 and 0.211, respectively) in the XR, and DOC is also not significantly related to HFOC and LF-N (p = −0.119 and −0.368, respectively) in the XR. Furthermore, moisture content in XRCW are positively associated with LFOC, HFOC, LF-N, and HF-N, while that in XR are positively associated with LFOC, DOC, LF-N, and HF-N. For the XRCW, bulk density is negatively connected with LFOC, HFOC, LF-N, and HF-N. For both XR and XRCW, there are no significant relationships between C, N and pH (p = > 0.05).

**Table 3:** Pearson correlation analysis among the factors in Xinxue River.

r	LFOC	HFOC	DOC	LF-N	LF-C/N	HF-N	HF-C/N	Moisture content	Bulk density
LFOC	1								
HFOC	0.564**	1							
DOC	−0.410*	−0.119	1						
LF-N	0.852**	0.715**	−0.368	1					
LF-C/N	0.575**	0.130	−0.201	0.190	1				
HF-N	−0.363	0.211	0.528**	−0.191	−0.227	1			
HF-C/N	0.693**	0.329	−0.621**	0.534**	0.417*	−0.665**	1		
Moisture content	−0.495*	−0.292	0.453*	−0.540**	−0.124	0.730**	−0.755**	1	
Bulk density	−0.118	0.186	0.090	0.025	−0.249	−0.036	−0.331	−0.126	1
pH	−0.315	−0.252	0.046	−0.380	−0.266	0.055	−0.122	0.356	−0.131

** Correlation is significant at the 0.01 level (2-tailed).

* Correlation is significant at the 0.05 level (2-tailed).

**Table 4:** Pearson correlation analysis among the factors in Xinxue River Constructed Wetland.

r	LFOC	HFOC	DOC	LF-N	LF-C/N	HF-N	HF-C/N	Moisture content	Bulk density
LFOC	1								
HFOC	0.371*	1							
DOC	−0.394*	0.393*	1						
LF-N	0.966**	0.404*	−0.396*	1					
LF-C/N	−0.257	−0.276	0.219	−0.428**	1				
HF-N	0.464**	0.865**	0.373*	0.513**	−0.328	1			
HF-C/N	−0.226	−0.106	−0.218	−0.256	0.082	−0.552**	1		
Moisture content	0.359*	0.780**	0.322	0.404*	−0.213	0.684**	−0.172	1	
Bulk density	−0.364*	−0.738**	−0.265	−0.428**	0.261	−0.703**	0.243	−0.950**	1
pH	0.189	0.304	−0.111	0.178	−0.107	0.189	0.225	0.167	−0.157

** Correlation is significant at the 0.01 level (2-tailed).

* Correlation is significant at the 0.05 level (2-tailed).

## Discussion

### Distribution of LFOC

LFOC, a type of labile OC, is easily affected by temperature, microbial activity, and conditions in the surrounding soils and sediments [40, 41]. LFOC consists of lipids, lignin monomers and dimers, and alky-aromatic compounds, which mostly originate from the degradation of plant material [42]. XRCW has much higher vegetation coverage and slower water velocity than XR, the insignificant differences in LFOC between XR and XRCW may suggest that LFOC levels are not significantly controlled by vegetation. This result was also suggested in the study of Yang et al [40], which focused on forest soils in China. In this study, LFOC is regarded to represent the carbon accumulation rate at a given time (without any consideration for carbon mineralization). So the significantly higher level of LFOC in upstream locations than that in downstream locations (for both wetlands types) suggests that the upstream wetland areas have higher carbon accumulation rates, especially in the XRCW.

Previous work has shown that LFOC can be partially transformed into HFOC during the process of carbon sequestration, while stable carbon can be transformed into DOC during the process of carbon decomposition and mineralization [42, 43]. With this in mind, the significant correlations between LFOC, HFOC, and DOC in the two wetlands may suggest the interactions of the different OC types [41]. The significant correlation between LFOC and LF-N in XR, and between LFOC and LF-N and HF-N in XRCW may also indicate that the OC and N of organic matter are supplementary to each other in soils and sediments of the two wetlands. Based on the study of Compton and Boone on nitrogen transformation [44], our results confirm that C and N share common sources for the accumulation of organic matter.

### Distribution of HFOC

The pattern and composition of HFOC are more stable than those of LFOC and DOC [15], which may be the main reason why the distribution of HFOC shows no significant difference between the two depths, the two location types, or the two wetland types. However, the significant differences in HFOC between wetland types and depths, and between depths and locations, demonstrate that HFOC does in fact different between the two wetland types.

The opposite variation trend of HFOC in depth of 15–25 cm at the two wetlands (Fig 3; Table 1) may reflect their different carbon accumulation rate. HFOC accumulation rate for XR is not significant between the two locations and so is HFOC accumulation rate in 0–15 cm depth of XRCW (Fig 3). The variations in HFOC at the two depths of XRCW may reflect background levels of carbon storage in the farmland on which the XRCW was created. Furthermore, the incremental accumulation rate of HFOC in XRCW may also indicate that the accumulation of HFOC is favorable with this kind of land use transformation. This finding is similar to Angers and Eriksen-Hamel [45], which reported on the conversion between no-tillage and full-tillage. The variations in HFOC between upstream and downstream locations are not significant in both wetland types, while the variations of LFOC between the two locations are significant in both wetland types. These results may suggest that at least some carbon mineralization is occurring concurrently to carbon sequestration [46, 47]. However, the carbon decomposition rate also plays a role, with the production of LFOC from vegetation and microorganisms impacting on the accumulation of HFOC in both the XR and XRCW.

### Distribution of DOC

DOC, consisting of colloidal organic compounds, was regarded as the metabolic intermediate of the carbon decomposition and mineralization process [48, 49]. Every year, the mineralization of DOM is estimated to convert significant quantities of carbon to CO_2_ [28]. On this basis, we at first considered DOC a proxy for the carbon decomposition rate. The quantities of DOC in the XRCW are significantly higher than in the natural XR wetland (Fig 4), possibly influenced by the lower current speed [50] and higher water solubility [51], which should suggest that the XRCW has a higher carbon decomposition rate. However, the negative correlation between DOC and LFOC in both the XR and XRCW may suggest that in reality the carbon decomposition rate is inhibited in areas with a high carbon accumulation rate.

### Controls on OC distribution

Previous studies have shown that the OC content of soil or sediment is significantly associated with microbial activity during the decomposition of vegetation and nutritive materials [52–54], and other studies have also suggested that the physicochemical characteristics of soils (e.g., moisture content, bulk density and pH) are the major factors defining the input of OC [18, 55]. In our study, OC (including LFOC, HFOC, and DOC) are significantly related to N (LF-N and HF-N), but pH is not significantly related to C and N (Tables 3 and 4). Furthermore, the correlations between C, N, and moisture content are inconsistent. Therefore, at the XR and XRCW, biochemical mechanisms, and not physicochemical conditions, are likely the main factors influencing OC content of the sediments.

## Conclusions

According to the analyses of the spatial and vertical distributions of different types of organic carbon in the XRCW and XR wetland, Shandong Province, China, the efficiency of carbon accumulation is evaluated in two wetland types. LFOC in the upstream locations is significantly higher than that in the corresponding downstream locations. Mean values of LFOC, HFOC, and DOC are all slightly higher in the XRCW than in the XR, despite the lower background HFOC value in XRCW. The results of this study demonstrate that the constructed wetland is gradually accumulating OC of different types, with the level of accumulation dependent on the balance between carbon accumulation and carbon decomposition. In contrast, the river wetland has relatively stable levels of OC. Our study provides new insights into effective mitigation measures for greenhouse gas emissions and lays the foundations for further study on the mechanism of carbon sequestration in constructed wetlands.
